# Prognostic value and immunological role of AXL gene in clear cell renal cell carcinoma associated with identifying LncRNA/RBP/AXL mRNA networks

**DOI:** 10.1186/s12935-021-02322-y

**Published:** 2021-11-27

**Authors:** Yi Wang, Ye Tian, Shouyong Liu, Zengjun Wang, Qianwei Xing

**Affiliations:** 1 Department of Urology, Affiliated Hospital of Nantong University, No. 20 West Temple Road, Nantong, 226001 Jiangsu Province China; 2 Department of Urology, The First Affiliated Hospital of Nanjing Medical University, No. 300 Guangzhou Road, Nanjing, 210029 Jiangsu Province China

**Keywords:** AXL, Clear cell renal cell carcinoma, Immunity, Prognosis, Network

## Abstract

**Backgrounds:**

This article aimed to explore the prognostic and immunological roles of AXL gene in clear cell renal cell carcinoma (ccRCC) for overall survival (OS) and to identify the LncRNA/RBP/AXL mRNA networks.

**Methods:**

AXL-related gene expression matrix and clinical data were obtained from The Cancer Genome Atlas (TCGA) dataset and AXL-related pathways were identified by gene set enrichment analysis (GSEA). We performed univariate/multivariate Cox regression analysis to evaluate independent prognostic factors and the relationships between AXL and immunity were also investigated.

**Results:**

The outcomes of us indicated that the AXL mRNA expression was up-regulated in ccRCC samples and high expression of AXL was associated with worse OS in TCGA dataset (P < 0.01). Further external verification results from HPA, UALCAN, ICGC dataset, GSE6344, GSE14994, and qRT-PCR remained consistent (all P < 0.05). AXL was also identified as an independent prognostic factor for ccRCC by univariate/multivariate Cox regression analysis (both P < 0.05). A nomogram including AXL expression and clinicopathological factors was established by us and GSEA results found that elevated AXL expression was associated with the JAK-STAT, P53, WNT, VEGF and MAPK signaling pathways. In terms of immunity, AXL was dramatically linked to tumor microenvironment, immune cells, immune infiltration, immune checkpoint molecules and tumor mutational burden (TMB). As for its potential mechanisms, we also identified several LncRNA/RBP/AXL mRNA axes.

**Conclusions:**

AXL was revealed to play prognostic and immunological roles in ccRCC and LncRNA/RBP/AXL mRNA axes were also identified by us for its potential mechanisms.

**Supplementary Information:**

The online version contains supplementary material available at 10.1186/s12935-021-02322-y.

## Background

Renal cancer (RC) is a common malignant tumor diagnosed in the urinary system, with approximately 76,080 new cases and 13,780 new death in the United States, 2021 [1]. As the most common histologic subtype of renal cell carcinoma (RCC), clear cell renal cell carcinoma (ccRCC) accounts for 75% of all RCCs, based on its histological classifications [2]. When diagnosed, more than 30% of RCC patients were localized or metastatic cases [3]. After surgical resection, ccRCC patients’ 5-year survival rate is merely 20% [4]. Therefore, it is necessary to better understand its potential mechanisms and found a predictor of prognosis for ccRCC patients.

The AXL receptor tyrosine kinase (AXL), also known as ARK, UFO, JTK11, Tyro7, belongs to the family of receptor tyrosine kinases (RTKs) and this gene AXL was first cloned from human chronic myelogenous leukemia (CML) cells, located at chromosome 19q13.2, encoding a 140 KDa protein with transforming ability [5, 6]. A growing number of evidence indicated the AXL gene played an important role in tumor development, metastasis, stem cell phenotype, drug resistance, and prognosis [7–11]. Previous study had shown that the AXL gene could be a downstream effector of the tumor cell Epithelial-to-mesenchymal transitions (EMTs) required by the metastasis of breast cancer [12]. As for EMT, it is a process in which the epithelial cells lose epithelial properties and acquire mesenchymal features, which contributes to tumor invasion, metastasis, and resistance to therapy. EMT is usually defined by the loss of the expression of epithelial marker E-cadherin and the gain of the mesenchymal marker vimentin [13]. According to another study, AXL signaling mediated DNA double strand broke repair and therefore targeting AXL might enhance the efficacy of radiation therapy [14]. Hence, this study aimed to explore the prognostic and immunological roles of AXL gene in ccRCC for overall survival (OS). Besides, its related signaling pathways and associations with immunity were also analyzed. As for its potential mechanisms, the LncRNA/RNA binding protein (RBP)/AXL mRNA networks were constructed to provide some references for future researches.

## Materials and methods

### Data processing

AXL-related gene expression matrix and clinical data of ccRCC patients were obtained from The Cancer Genome Atlas (TCGA) data portal (http://cancergenome.nih.gov/), including 539 ccRCC tumor samples and 72 normal tissue samples. Ultimately, we extracted the gene profiles and clinical information for subsequent analysis after excluding cases lacking key clinical information. Moreover, another three ccRCC datasets from the Gene Expression Omnibus (GEO) database (GSE6344 and GSE14994; https://www.ncbi.nlm.nih.gov/geo/) and the International Cancer Genome Consortium database (ICGC; https://icgc.org/) were used as external verifications. Differently expressed genes (DEGs) were calculated by using the “Limma” R package. Besides, |log2 fold change (FC)|≥ 1 as well as adjusted P-values (FDR) < 0.05 were defined as the cut-off criteria.

### Screening of AXL expression and functional and pathway enrichment in ccRCC

By using “Limma” R package, DEGs between tumor tissue and adjacent non-tumor renal tissue was investigated and the expression of AXL in different gender, race, grade, T stage was also compared. Moreover, AXL-related pathways were identified by Gene set enrichment analysis (GSEA) [15].

### Quantitative real-time PCR (qRT-PCR)

We utilized six pairs of ccRCC tumor tissue and adjacent non-tumor renal tissue, acquired from the Affiliated Hospital of Nantong University, to verify the AXL mRNA expression. Detailed clinicopathological data of these six ccRCC patients were summarized in Additional file 1: Table S1. Age of these six ccRCC patients was ranged from 42 to 78; T stage was ranged from T1–T4; N stage was ranged from N0–N1; M stage was ranged from M0-M1; Stage was ranged from stage I–IV; Grade was ranged from grade 1–4. Total RNA from ccRCC tissues and adjacent normal tissues was acquired using TRIzol reagent (Invitrogen, Carlsbad, CA, USA) and cDNA was synthesized using HiScript III RT SuperMix for qPCR (+gDNA wiper) (Vazyme, Nanjing, China) on the basis of manufacturer’s protocol. The qRT-PCR was performed by using StepOne Plus Real-time PCR system (Applied Biosystems, Foster City, CA, USA) with ChamQ SYBR qPCR Master Mix (High ROX Premixed) (Vazyme). The relevant primers were presented as follows: AXL, F: 5'-GTGGGCAACCCAGGGAATATC-3', R: 5'-GTACTGTCCCGTGTCGGAAAG-3'; β-actin, F: 5'- ATGACTTAGTTGCGTTACACC-3', R: 5'-GACTTCCTGTAACAACGCATC-3'. ABI Step One Software version 2.1 was used for data analysis, and 2^−ΔΔCt^ methods were used to calculate the relative mRNA levels. This study was approved by the Institutional Research Ethics Committees of the Affiliated Hospital of Nantong University (Ethical code: 2019-L018).

### Verification of the AXL protein expression utilizing the HPA and UALCAN databases

By means of The Human Protein Atlas (HPA, http://www.proteinatlas.org/) online database, we validated the AXL protein expression in ccRCC by immunohistochemical staining. We also utilized the UALCAN website (http://ualcan.path.uab.edu/analysis-prot.html) to confirm the AXL expression between the primary ccRCC tumor and normal tissues by means of CPTAC dataset analysis.

### Univariate/multivariate Cox hazard regression analysis and nomogram establishment

Univariate/multivariate Cox hazard regression analysis were employed to the ccRCC patients from TCGA database by R package to identify whether eight clinicopathological parameters (race, gender, age, grade, T, M, N, stage) and AXL could be independent factors correlated with OS. The R “rms” package was also utilized by us to conduct a nomogram model to predict the likelihood of OS by integrating eight clinicopathological parameters (race, gender, age, grade, T, M, N, stage) and AXL.

### Microsatellite instability (MSI), tumor mutational burden (TMB), tumor neoantigen burden (TNB) and immunity evaluation

We utilized the single gene pan-cancer analysis tool provided by the Sangerbox website (http://www.sangerbox.com/tool) to evaluate the relationships between the AXL expression and MSI, TMB, TNB by the pearson’s method, with the threshold of P < 0.05 [16–18]. In terms of immunity, four aspects including immune cells pathway, tumor microenvironment, immune checkpoint molecules, tumor immune infiltration were involved. The single gene pan-cancer analysis tool provided by the Sangerbox website was also applied by us to explore the associations between the AXL expression and immune cells pathway, tumor microenvironment, immune checkpoint molecules, tumor immune infiltration by the spearman’s or the pearson’s method, with the threshold of P < 0.001 [19–21].

### Establishment of LncRNA/RBP/AXL mRNA networks

As the manner in previously published articles [22–24], starBase v2.0 (http://starbase.sysu.edu.cn/) was utilized by us to find RBP-AXL mRNA, RBP-LncRNA targets to identify LncRNA/RBP/AXL mRNA networks. Combined with the threshold of strict stringency (≥ 5) and pan-Cancer ≥ 10 cancer types in starBase v2.0, hub LncRNAs (|log2 FC|≥ 1, FDR < 0.05 and P value < 0.05) in TCGA ccRCC, LncRNAs positively correlated with AXL (correlation coefficient ≥ 0.3 and P value < 0.001) in TCGA ccRCC, the LncRNA/RBP/AXL mRNA networks were established and visualized by Cytoscape 3.6.1 software. Therein, hub LncRNAs in TCGA ccRCC were defined as that LncRNAs were differently expressed (|log2 FC|≥ 1 and FDR < 0.05) and significantly associated with OS (P value < 0.05).

## Results

### The expression and prognosis of AXL in ccRCC

**Fig. 1:**
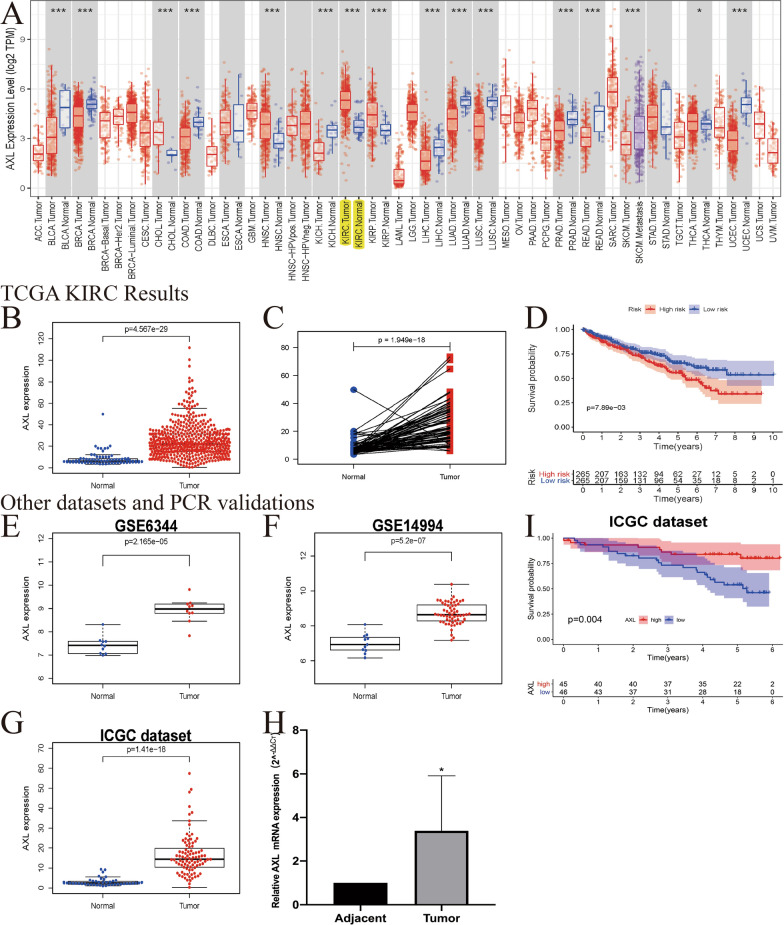
The expression and prognosis of AXL in ccRCC; **A** the expression of AXL in various cancers by using TCGA database; **B** relative expression levels of the AXL expression between the ccRCC and normal tissues in TCGA dataset (N = 72; T = 539); **C** pairwise boxplot of the AXL expression between the matched ccRCC and normal tissues in TCGA dataset (N = 72; T = 72); **D** Kaplan–Meier curves in the TCGA database; **E** Boxplot of the AXL expression in GSE6344 (N = 10; T = 10); **F** boxplot of the AXL expression in GSE14994 (N = 11; T = 59); **G** Boxplot of the AXL expression in the ICGC dataset (N = 45; T = 91); **H** qRT-PCR results (N = 6; T = 6); **I** Kaplan–Meier curves in the ICGC dataset

We investigated the mRNA expression levels of AXL to identify differential expression patterns between tumor tissue and adjacent non-tumor renal tissue in TCGA pan-cancers (Fig. 1A). Compared with the normal tissue, the AXL expression was significantly increased in ccRCC tumor tissue (P < 0.001, Fig. 1B). Pairwise boxplot also suggested that the AXL mRNA in tumor samples could have a higher expression (P < 0.001, Fig. 1C). Moreover, we classified ccRCC patients into low- and high-risk subgroups by the AXL median expression. Kaplan–Meier curve showed that patients in the high-AXL groups had worse OS than those in the low-AXL groups (P < 0.05; Fig. 1D). We further utilized ICGC dataset, GSE6344, GSE14994 and qRT-PCR as external verifications, to validate the AXL mRNA expression. Consistent with TCGA results, AXL was up-regulated in ccRCC tumor tissues and related to significant OS (all P < 0.05; Fig. 1E–I). Detailed clinicopathological data of these six ccRCC patients utilized by qRT-PCR were summarized in Additional file 1: Table S1. All of these indicated that the AXL mRNA expression was up-regulated in ccRCC samples and it was significantly associated with OS.

### External verification of AXL protein expression in ccRCC

**Fig. 2:**
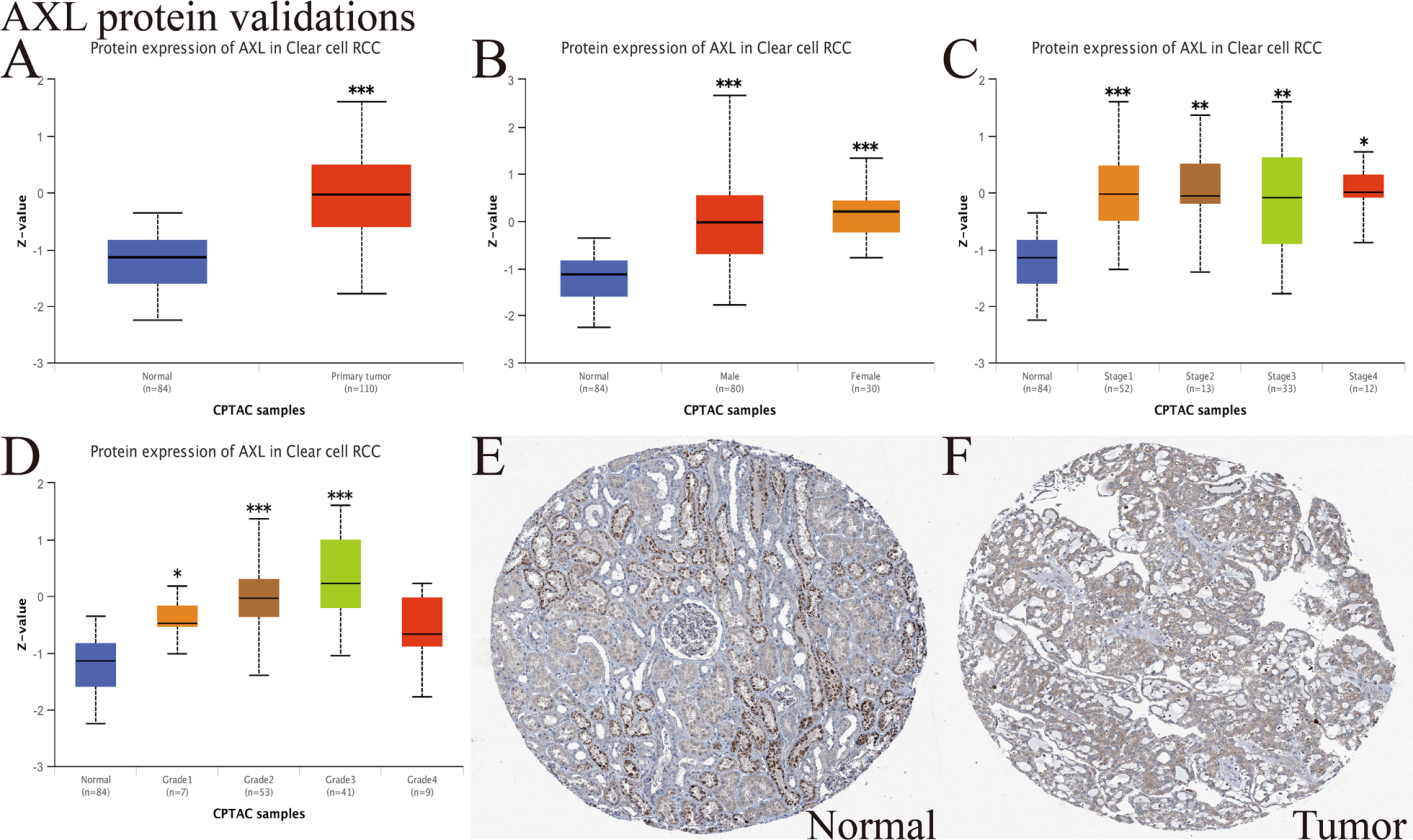
External verification of AXL protein expression in ccRCC; **A** the AXL protein expression distribution in ccRCC primary tumor and normal tissues; **B** the AXL protein expression distribution in different genders; **C** the AXL protein expression distribution in different stages; **D** the AXL protein expression distribution in different grades; **E**–**F** The immunohistochemical staining from the HPA database indicated that AXL was highly expressed in tumor tissues; Compared with the normal groups, *P < 0.05; **P < 0.01; ***P < 0.001

The UALCAN website was utilized to validate the AXL protein expression by CPTAC analysis. As displayed in Fig. 2A, it presented that the AXL total protein expression was elevated in primary tumor tissue than in adjacent non-tumor renal tissue (P < 0.001). Figure 2B–D presented the AXL protein expression distribution in different genders, stages or grades, respectively. Moreover, immunohistochemical staining of the HPA database (https://www.proteinatlas.org/) suggested that AXL had a higher expression in ccRCC tumor tissue compared with adjacent non-tumor renal tissue (Fig. 2E, F). These results further confirmed that the AXL protein expression was also elevated in ccRCC samples than in adjacent non-tumor renal tissues, consistent with its mRNA expression.

### Relationships between AXL expression and clinicopathologic characteristics

**Fig. 3:**
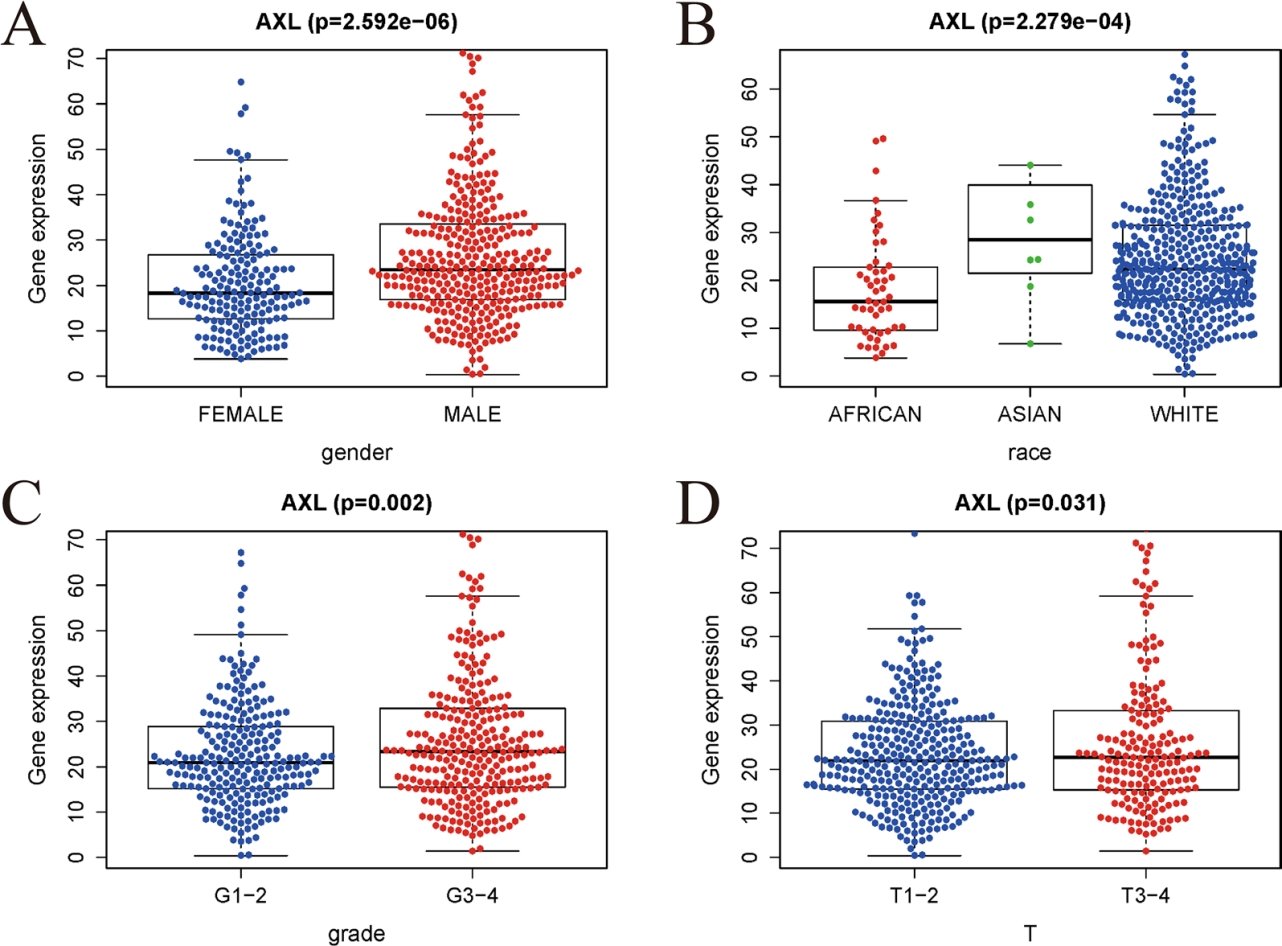
Association with AXL expression and clinicopathologic characteristics; **A** gender; **B** race; **C** grade; **D** T stage

We used logistic regression analysis to evaluate the relationships between the AXL expression and clinicopathologic characteristics in ccRCC patients. As detailed in Fig. 3, significant associations were displayed between the AXL expression and gender, race, grade, T stage (all P < 0.05). Obviously, low expression of AXL was significantly related to G1–2 and T1–2. In other words, ccRCC patients with elevated AXL expression were prone to have an advanced form of this disease than those with low AXL expression.

### AXL could serve as an independent prognostic factor and nomogram establishment

**Fig. 4:**
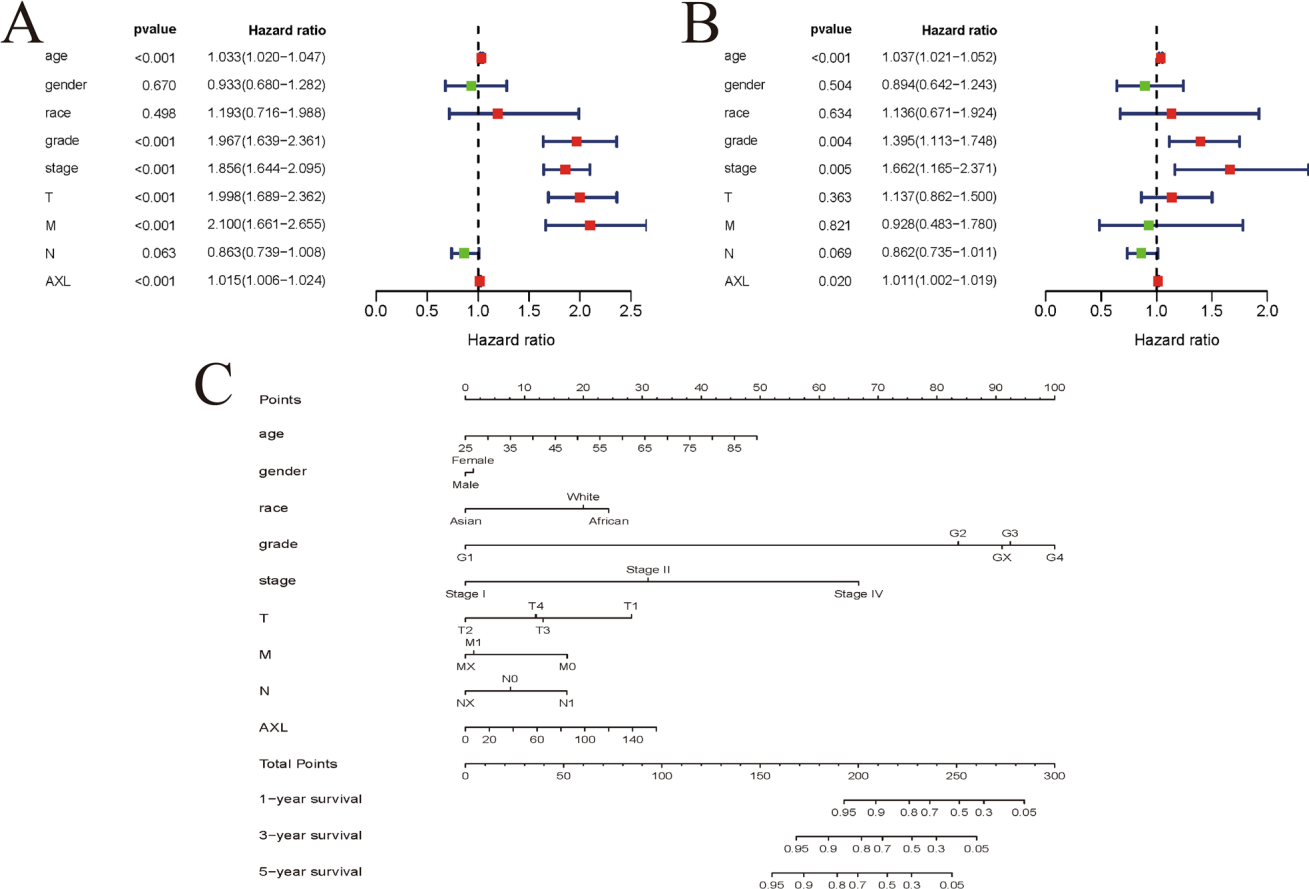
AXL could serve as an independent prognostic factor and established nomogram; **A**, **B** Univariate and multivariate Cox regression analysis of clinicopathologic variables and AXL of ccRCC patients in TCGA database; **C** developed nomogram to predict the overall survival of ccRCC patients based on AXL expression and clinicopathologic parameters

Univariate/multivariate Cox regression analyses were performed on data obtained from the TCGA dataset to evaluate whether AXL could serve as an independent factor related to OS (Table 1). In the univariate Cox analysis, age (HR = 1.033), grade (HR = 1.967), pathological stage (HR = 1.856), T stage (HR = 1.998), metastasis (HR = 2.100) and the AXL expression (HR = 1.015) were significantly related to the OS (all P < 0.001; Fig. 4A). In the multivariate Cox analysis, age (HR = 1.037), grade (HR = 1.395), stage (1.662 = 4.10) and the AXL expression (HR = 1.011) were markedly linked to the OS (all P < 0.05; Fig. 4B). Taken together, the above results suggested that AXL expression could be an independent predictor of prognosis for ccRCC. We also utilized the R “rms” package to conduct a nomogram model to predict the likelihood of OS by integrating eight clinicopathological parameters (race, gender, age, grade, T, M, N, stage) and AXL (Fig. 4C). All in all, AXL was identified as an independent prognostic factor for ccRCC by univariate/multivariate Cox regression analysis, and moreover, a nomogram including AXL expression and clinicopathological factors was also established by us to intuitively predict 1-, 3-, 5-year OS of ccRCC patients.

**Table 1:** Associations with overall survival and clinicopathologic characteristics in TCGA patients using univariate and multivariate cox analysis

Clinical characteristics	Univariate analysis		Multivariate analysis	
	HR (95% CI)	p-value	HR (95% CI)	p-value
Age	1.033 (1.020–1.047)	** < 0.001**	1.037 (1.021–1.052)	** < 0.001**
Gender	0.933 (0.680–1.282)	0.670	0.894 (0.642–0.243)	0.504
Race	1.193 (0.716–1.988)	0.498	1.136 (0.671–1.924)	0.634
Grade	1.967 (1.639–2.361)	** < 0.001**	1.395 (1.113–1.748)	**0.004**
Stage	1.856 (1.644–2.095)	** < 0.001**	1.662 (1.165–1.371)	**0.005**
T	1.998 (1.689–2.362)	** < 0.001**	1.137 (0.862–1.500)	0.363
M	2.100 (1.661–2.655)	** < 0.001**	0.928 (0.483–0.780)	0.821
N	0.862 (0.739–1.008)	0.063	0.862 (0.735–0.011)	0.069
AXL	1.015 (1.006–1.024)	** < 0.001**	1.011 (1.002–1.019)	**0.020**

### AXL-related signaling pathways identified by GSEA

**Fig. 5:**
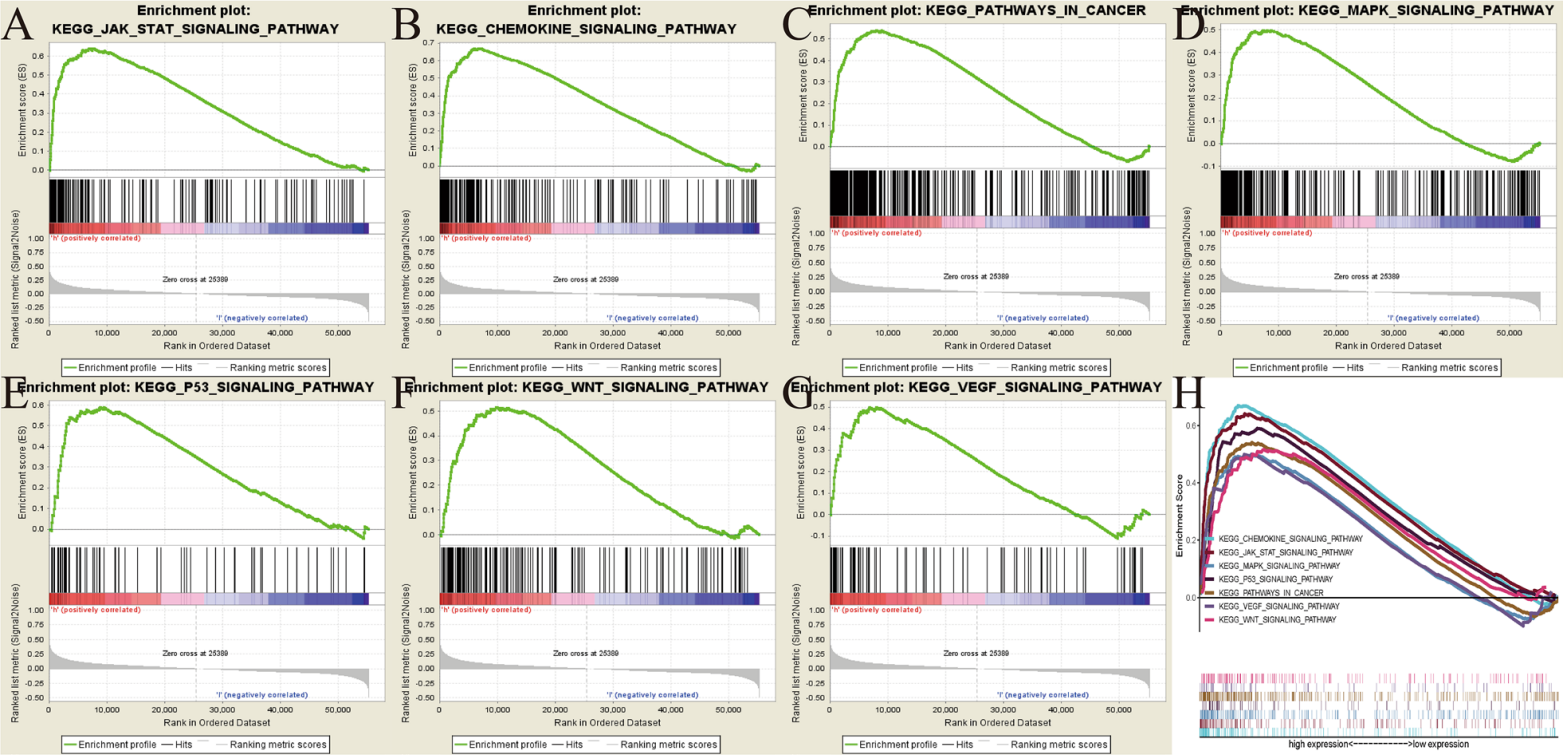
Enrichment plots from gene set enrichment analysis (GSEA); **A** JAK-STAT signaling pathway; **B** Chemokine signaling pathway; **C** pathway in cancer; **D** MAPK pathway; **E** p53 signaling pathway; **F** Wnt signaling pathway; **G** VEGF signaling pathway; **H** The seven most significantly enriched signaling pathways based on their normalized enrichment score and the expression map

GSEA was performed between the high- and low-AXL expression matrixes to identify AXL-related signaling pathways and seven pathways that exhibited significantly differential enrichment in the high-AXL expression phenotype, were finally identified and selected, including the JAK-STAT, P53, WNT, VEGF and MAPK signaling pathways (Fig. 5 and Table 2). All of these results indicated the potential signaling pathways related to AXL gene in ccRCC, offering help in better understanding the pathogenesis underlying this disease.

**Table 2:** Gene sets enriched in phenotype high;

MSigDB collection	Gene set name	NES	NOM p-val	FDR q-val
c2.cp.kegg.v7.1.symbols.gmt	JAK_STAT_SIGNALING_PATHWAY	2.703	< 0.001	< 0.001
	CHEMOKINE_SIGNALING_PATHWAY	2.562	< 0.001	< 0.001
	PATHWAYS_IN_CANCER	2.340	< 0.001	0.001
	MAPK_SIGNALING_PATHWAY	2.257	< 0.001	0.001
	P53_SIGNALING_PATHWAY	2.133	< 0.001	0.003
	WNT_SIGNALING_PATHWAY	2.101	0.006	0.004
	VEGF_SIGNALING_PATHWAY	2.090	< 0.001	0.004

### Associations between AXL and PPI, MSI, TMB, TNB in ccRCC

**Fig. 6:**
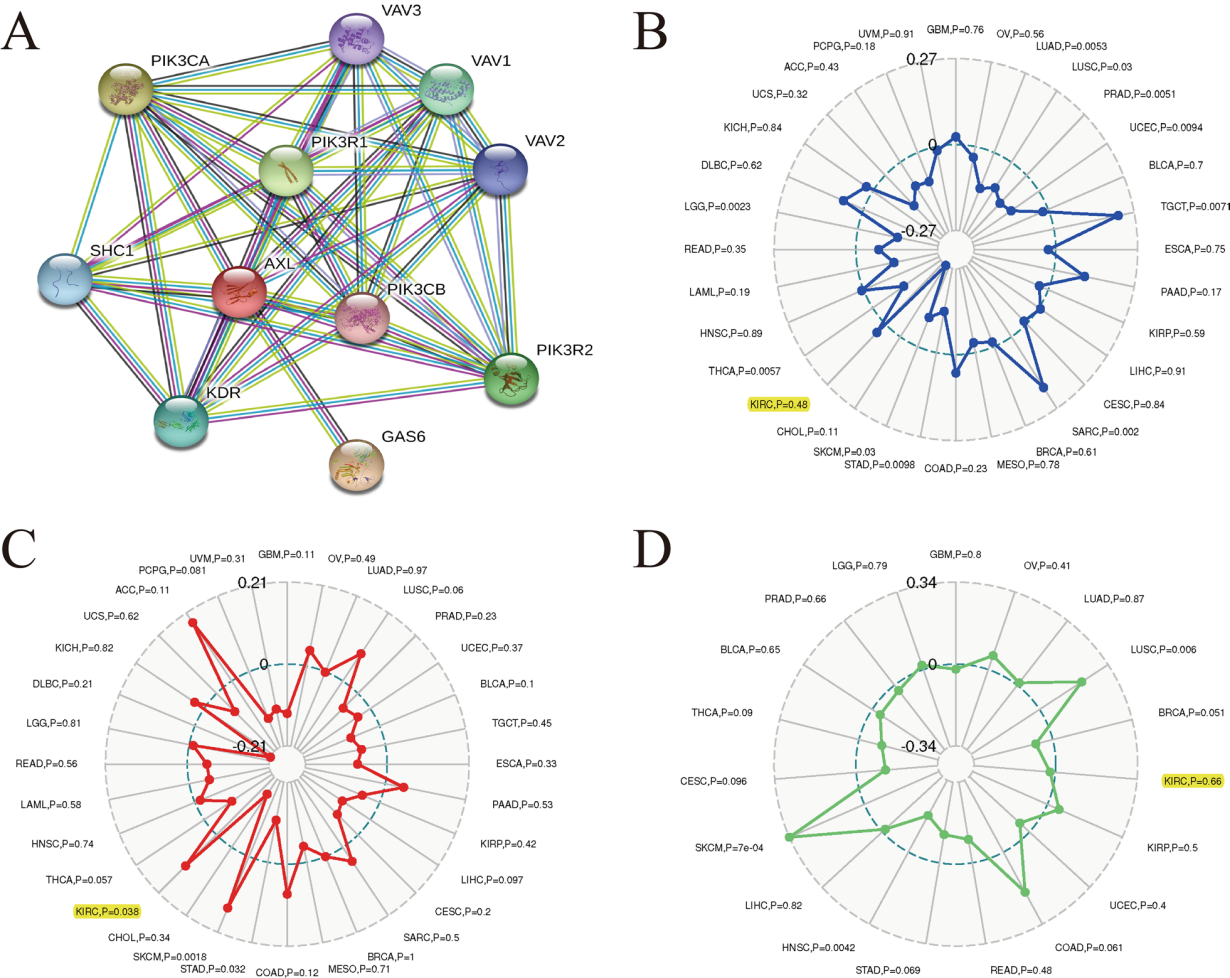
Associations between AXL and PPI, MSI, TMB, TNB in ccRCC; **A** PPI network; **B** Associations between AXL and MSI; **C** Associations between AXL and TMB; **D** Associations between AXL and TNB

PPI network indicated that ten genes (VAV1, VAV2, VAV3, PIK3CA, PIK3CB, PIK3R1, PIK3R2, SHC1, KDR and GAS6) were significantly relevant with AXL expression (Fig. 6A). We utilized the Sangerbox website (http://www.sangerbox.com/tool) to evaluate the relationships between the AXL expression and MSI, TMB, TNB by the pearson’s method, with the threshold of P < 0.05. Our results shed light on that AXL was significantly associated with TMB (P = 0.038) in ccRCC, while it was not related to MSI (P = 0.48) or TNB (P = 0.66) (Fig. 6B-D). Currently, MSI, TMB and TNB had been regarded as vital biomarkers of various cancers for predicting prognosis or immune responses [25, 26]. As revealed in this article, AXL was significantly related to TMB in ccRCC, based on our results.

### Associations between AXL and the immune infiltrations, tumor microenvironment, methyltransferase in ccRCC

**Fig. 7:**
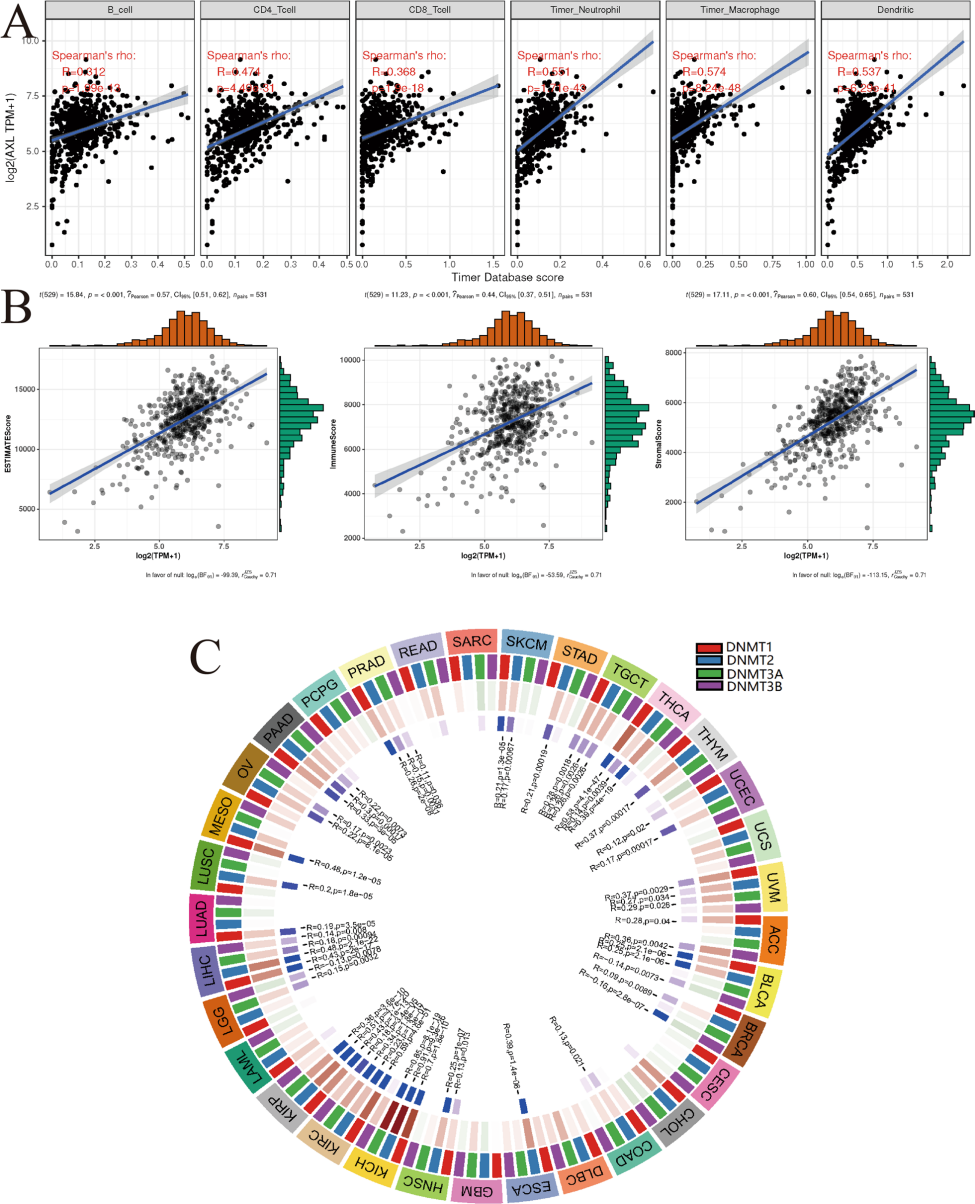
Associations between AXL and the immune infiltrations, tumor microenvironment, methyltransferase in ccRCC; **A** associations between AXL and immune infiltrations; **B** associations between AXL and immune microenvironment including immune cells, stromal cells and both of them. **C** Associations between AXL and methyltransferase

By the pearson’s method, we explored the associations between the AXL expression and immune infiltrations by the Sangerbox website and found the AXL expression was significantly related to CD4^+^ T cell infiltration, B cell infiltration, neutrophil infiltration, CD8^+^ T cell infiltration, dendritic cell infiltration and macrophage infiltration (all P < 0.001; Fig. 7A). Besides, it indicated that AXL has significant relationships with immuneScore, stromalScore and ESTIAMTEScore (all P < 0.001; Fig. 7B). As showed in Fig. 7C, four methyltransferase including DNMT1, DNMT2, DNMT3A and DNMT3B, were all significantly associated with the AXL expression in various tumors, especially in ccRCC (all P < 0.001). All of these results indicated that the AXL expression was significantly associated with immune infiltration, tumor microenvironment, methyltransferase in ccRCC.

### Associations between AXL and mismatch repair proteins, immune cells, immune checkpoint molecules

**Fig. 8:**
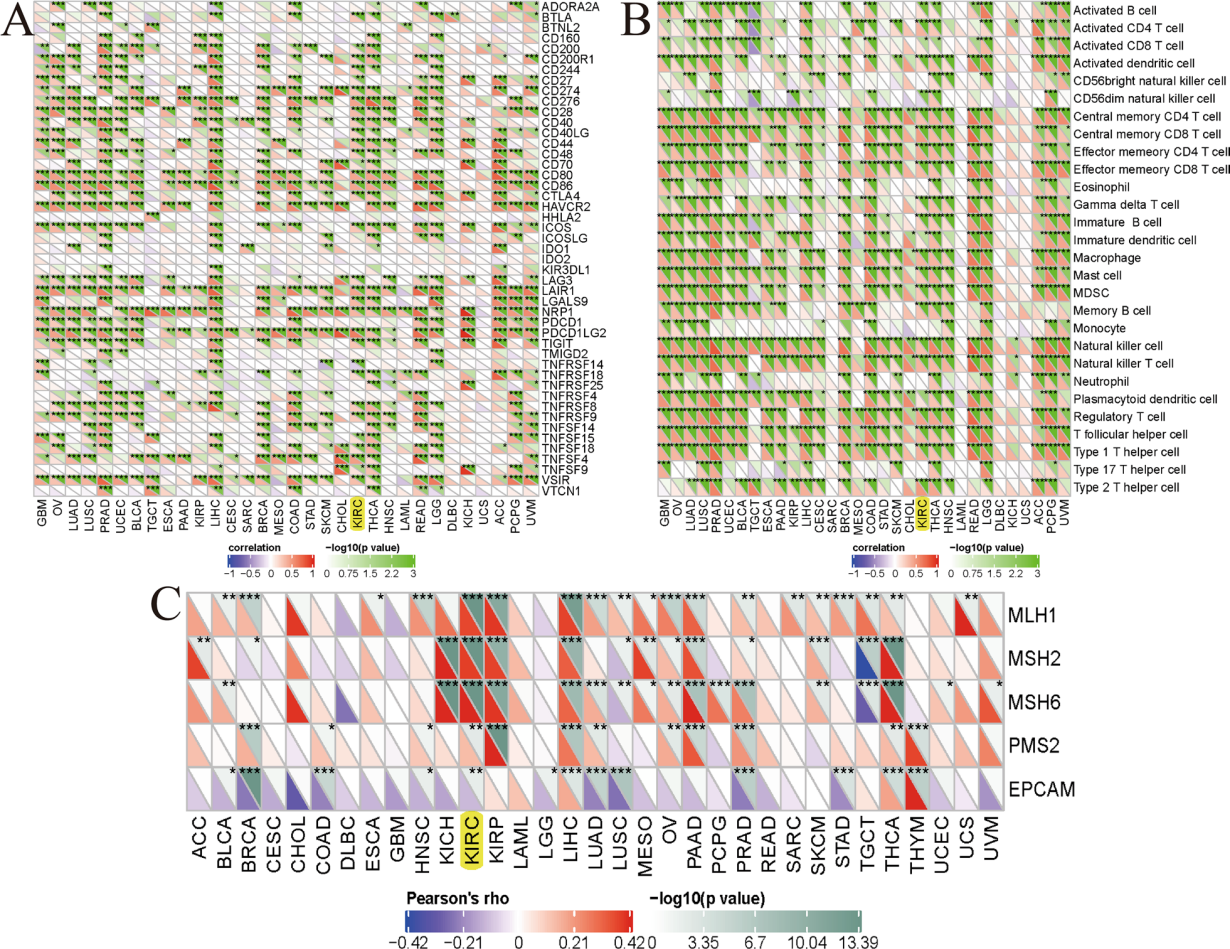
Associations between AXL and immune checkpoint molecules, immune cells, mismatch repair proteins; **A** associations between AXL and immune checkpoint molecules; **B** associations between AXL and immune cells; **C** associations between AXL and mismatch repair proteins; *P < 0.05; **P < 0.01; ***P < 0.001

To further investigate the correlations between AXL and immunity in ccRCC tissues from the TCGA database, we found that AXL was significantly associated with the immune checkpoint molecules like BTLA, CD244, CD274, CTLA4 etc. in ccRCC (all P < 0.05; Fig. 8A). Moreover, we studied the associations between AXL and the immune cells and found that the AXL was significantly linked to immune cells, containing Actived CD4 T cell, Actived CD8 T cell, Actived memory B cell etc. ((all P < 0.05; Fig. 8B). Mismatch repair proteins including MLH1, MSH2, MSH6, PMS2 and EPCAM, also displayed significant associations with AXL in ccRCC (all P < 0.01; Fig. 8C). All of these results also indicated that the AXL expression was dramatically linked to mismatch repair proteins, immune cells, immune checkpoint molecules in ccRCC.

### Establishment of LncRNA/RBP/AXL mRNA networks

**Fig. 9:**
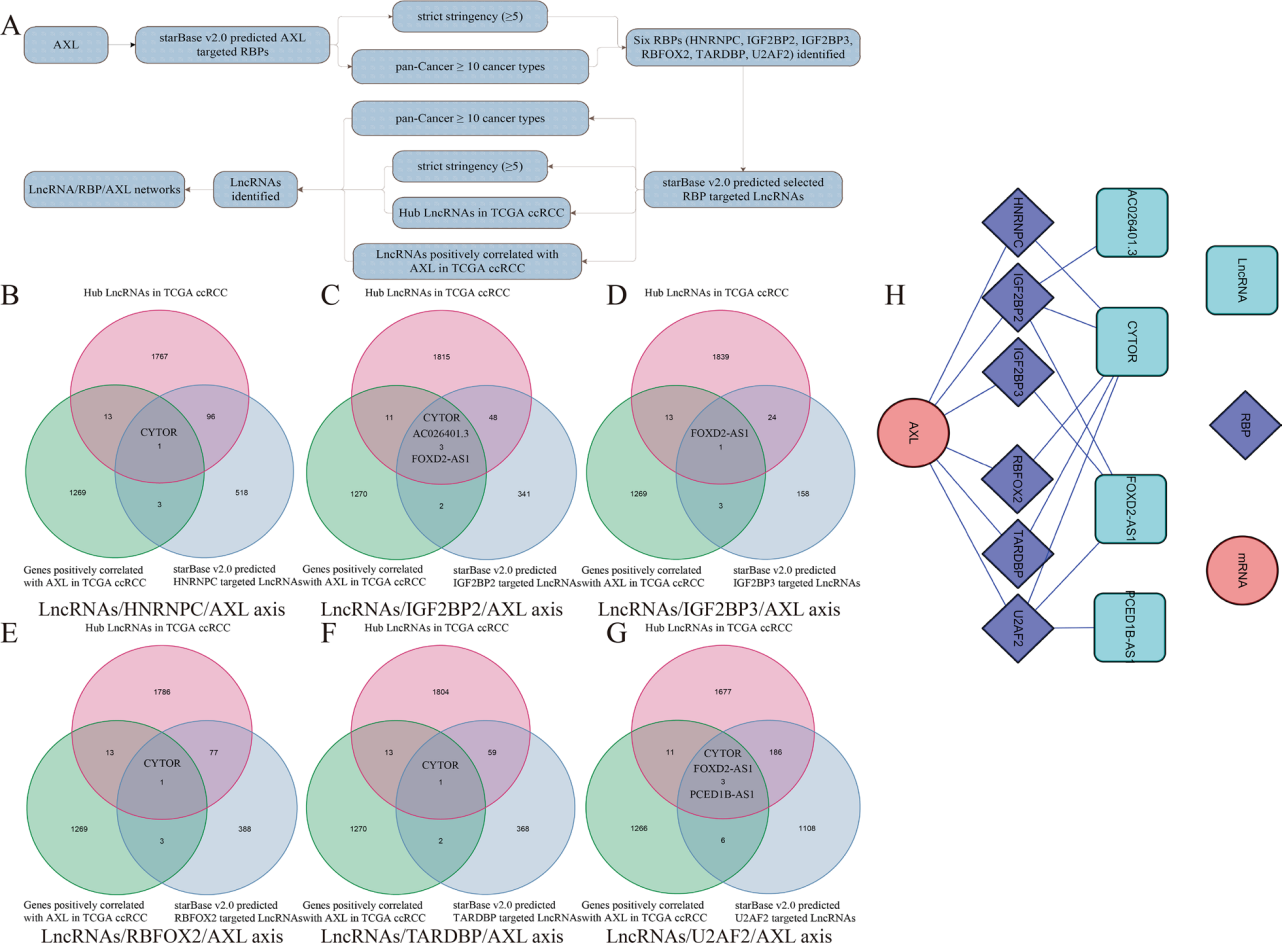
Establishment of LncRNA/RBP/AXL mRNA networks; **A** the whole workflow of identifying LncRNA/RBP/AXL mRNA networks; **B** Venn diagrams of identifying the LncRNAs/HNRNPC/AXL axes; **C** Venn diagrams of identifying the LncRNAs/IGF2BP2/AXL axes; **D** Venn diagrams of identifying the LncRNAs/IGF2BP3/AXL axes; **E** Venn diagrams of identifying the LncRNAs/RBFOX2/AXL axes; **F** Venn diagrams of identifying the LncRNAs/TARDBP/AXL axes; **G** Venn diagrams of identifying the LncRNAs/U2AF2/AXL axes; **H** the LncRNA/RBP/AXL mRNA networks

As for RBPs, they acted through a wide range of mechanisms, including mRNA stability, polyadenylation, alternative splicing, mRNA localization and translation [27, 28] and we mainly utilized its function of mRNA stability in this article. As the manner in previously published articles [22–24], LncRNA-RBP interactions on regulating mRNA stability had been fully and increasingly elucidated. The whole workflow of identifying LncRNA/RBP/AXL mRNA networks was detailed in Fig. 9A. To find the upstream of AXL, starBase v2.0 was utilized to find AXL-RBP targets with the threshold of strict stringency (≥ 5) and pan-Cancer ≥ 10 cancer types. Six possible RBPs including HNRNPC, IGF2BP2, IGF2BP3, RBFOX2, TARDBP and U2AF2 were identified. To find the upstream of a selected RBP, starBase v2.0 was utilized to find RBP-LncRNA targets, with the consideration of the threshold of strict stringency (≥ 5) and pan-Cancer ≥ 10 cancer types in starBase v2.0, hub LncRNAs (|log2 FC|≥ 1, FDR < 0.05 and P value < 0.05) in TCGA ccRCC, LncRNAs positively correlated with AXL (correlation coefficient ≥ 0.3 and P value < 0.001) in TCGA ccRCC. Therein, hub LncRNAs in TCGA ccRCC were defined as that LncRNAs were differently expressed (|log2 FC|≥ 1 and FDR < 0.05) and significantly associated with OS (P value < 0.05). Venn diagrams of identifying the LncRNAs/HNRNPC/AXL axes, the LncRNAs/IGF2BP2/AXL axes, the LncRNAs/IGF2BP3/AXL axes, the LncRNAs/RBFOX2/AXL axes, the LncRNAs/TARDBP/AXL axes, the LncRNAs/U2AF2/AXL axes, were showed in Fig. 9B–G, respectively. Taken together, the LncRNA/RBP/AXL mRNA networks had been successfully established and presented in Fig. 9H. All in all, we identified several LncRNA/RBP/AXL mRNA axes by means of bioinformatics analysis to reveal the potential mechanisms of AXL in ccRCC. Further validation experiments were still required to verify these axes in our subsequent articles.

## Discussion

As the most common histological subtype of RCC, ccRCC is resistant to radiotherapy and chemotherapy. About 30% of RCC patients would be detected with metastatic lesions at the time of primary diagnosis [29]. Nearly one-third of these patients treated with partial or radical nephrectomy finally developed metastatic RCC [4]. AXL is a tyrosine protein kinase receptor protein coding gene of the TAM family. Recent studies have revealed that the AXL roles in tumorigenesis and development in a variety of tumors [30]. However, few studies focused on the prognostic roles of AXL in ccRCC.

In our study, AXL was found to be highly expressed in patients with ccRCC and significantly correlated with clinicopathologic parameters, associated with a poor prognosis of the OS. Logistic regression analysis indicated that increased expression of AXL in ccRCC was significantly associated with gender, race, grade, T stage. In addition, univariate/multivariate Cox regression analyses suggested AXL gene could be an independent predictor of prognosis for ccRCC. Accumulating evidence had presented that AXL expression was correlated with poor prognosis or metastasis in various cancer types. As for breast cancer, AXL predicted poor overall survival and was a crucial EMT-induced regulator of breast cancer metastasis [12]. Therein, EMT is a process in which the epithelial cells lose epithelial properties and acquire mesenchymal features, which contributes to tumor invasion, metastasis, and resistance to therapy. It is usually defined by the loss of the expression of epithelial marker E-cadherin and the gain of the mesenchymal marker vimentin [13]. Another study had identified AXL as a major regulator of intrinsic and chemotherapy-induced invasion. In early colorectal cancer (CRC), AXL was found to be a biomarker of poor prognosis [31]. Similar as our results, AXL was markedly related to higher pathological grade and shorter progression-free survival in head and neck squamous cell carcinoma (HNSCC) and its overexpression could increase the resistance of HNSCC to radiotherapy, chemotherapy and cetuximab [14]. AXL expression was significantly correlated with multiple clinicopathological characteristics of tumor invasiveness, and could predict poor prognosis in hepatocellular carcinoma (HCC) patients after partial hepatectomy [32].

To further investigate the AXL associated signaling pathways, GSEA was performed between the high- and low-AXL expression matrixes by using TCGA data. A total of seven related pathways were finally identified with significantly differential enrichment in the high-AXL expression phenotype, including the JAK-STAT, P53, WNT, VEGF and MAPK signaling pathways. These pathways are widely involved in oncogenesis and development of ccRCC. Among the pathways mentioned above, it is well known that the carcinogenesis and development of RCC are most closely related to the activation of the VEGF signaling pathway and a variety of VEGF-targeted therapies had been developed and approved [33–35]. In our previous study, we demonstrated that fructose-bisphosphate aldolase A (ALDOA) may induce metastasis and cell proliferation via the Wnt/β-catenin pathway in RCC [36]. Previous study presented that the lack of proteins expression in the JAK-STAT pathway was highly related to resistance of RCC to IFN-α, while restoring JAK or STAT1 expression might help improve the response rate of RCC to IFN-α [37]. A study suggested that the expression of HSPB7 plays a role in the p53 pathway, and its downregulation by hypermethylation may play an important role in renal tumorigenesis [38]. Targeted drugs, especially tyrosine kinase inhibitors (TKI), could significantly improve the clinical results of patients with advanced RCC. Therein, cabozantinib, as an oral TKI targeting VEGFR, MET, RET and AXL, was notably more effective than everolimus in the second-line setting of patients with ccRCC [39].

In terms of immunity, AXL was found to be significantly related to tumor microenvironment, immune checkpoint molecules and immune infiltration by our results, indicating that AXL might play vital roles in immunity. With the rapid progress of immunotherapy, the results of relevant clinical trials on RC patients had been published with impressive positive results. Moreover, phase III clinical studies on immunocheckpoint inhibitors had been carried out in succession, and the roles of immunotherapy had gradually became prominent. As reported by Cattrini et al. [40], AXL was significantly correlated with BTLA, CD244, CD274, CTLA4 and other immune checkpoint molecules in ccRCC, indicating the vital roles of AXL in immunotherapy.

Lots of non-coding RNAs, especially LncRNAs and microRNAs, had been reported that they played a key role in regulating various biological and pathological processes, such as proliferation, cell cycle, apoptosis, invasion, migration, metastasis and drug resistance, by regulating their target mRNAs transcriptionally or posttranscriptionally [41]. Numerous studies highlighted the role of lncRNA-miRNA-mRNA axis in RCC. Qu et al. [42] demonstrated that lncARSR facilitated the expression of c-MET and AXL in RCC cells by competitively binding miR-34/miR-449 to promote sunitinib resistance. Zhang et al. [43] demonstrated that lncRNA maternally expressed gene 3 (MEG3) caused cholestasis by destabilizing Shp via serving as a guide RNA scaffold to recruit PTBP1 to Shp mRNA. As a hotspot in cancer research, RBPs had been studied in various cancers and the LncRNA/RBP/mRNA mechanisms had also been revealed [44, 45]. As for RBPs, they acted through a wide range of mechanisms, including mRNA stability, polyadenylation, alternative splicing, mRNA localization and translation [27, 28] and we mainly utilized its function of mRNA stability in this article. As the manner in previously published articles [22–24], LncRNA-RBP interactions on regulating mRNA stability had been fully and increasingly elucidated. Zou et al. [23] found that LINC00324 could promote cell proliferation via binding with HuR and stabilizing FAM83B expression in gastric cancer. Zhang et al. [22] shed light on that MYC up-regulated LINC00319 contributed to AML leukemogenesis through stabilizing SIRT6 mRNA. In this article, we also successfully established and identified several possible LncRNA/RBP/AXL mRNA axes, to reveal the potential mechanisms of AXL in ccRCC. More basic researches were needed to verify these axes and to further explore the molecular mechanisms of AXL in ccRCC.

It was the first time for us to explore the comprehensive roles of AXL in ccRCC with a robust statistical approach and we not only analyzed the AXL mRNA expression, but also verified its protein expression. Moreover, the ICGC, GEO, HPA datasets and qRT-PCR results were utilized as external verifications, making our results more persuasive. Moreover, we also emphasized the role of immunity and found that AXL was significantly associated with tumor microenvironment, immune checkpoint molecules, immune infiltration and TMB. Last but not least, several LncRNA/RBP/AXL mRNA axes were identified for potential mechanisms. Our findings were anticipated to provide new insights of AXL in ccRCC for future work. Due to the limitations of the study design, the study failed to clarify the correlations between the expression of AXL mRNA and the expression of AXL protein in RCC. Moreover, the small sample size of evaluated normal renal tissue samples in both TCGA and ICGC databases might lead to distortion of conclusions. Therefore, more normal samples were needed to correct our results. In addition, clinical information on the use of TKI or immunotherapy, whether the initial diagnosis was metastatic, whether there was recurrence after nephrectomy, etc., could not be retrieved from the online database. The absence of the important information that directly affects the final survival outcome may lead to an inaccurate assessment. Last but not least, we identified several LncRNA/RBP/AXL mRNA axes for potential mechanisms in this study. Given space limitation, the validation experiments of these LncRNA/RBP/AXL mRNA networks were not currently included in this article and it would be conducted in our subsequent articles.

## Conclusions

Taken together, our results indicated that AXL was revealed to play prognostic and immunological roles in ccRCC. Moreover, five primary pathways including the JAK-STAT, P53, WNT, VEGF and MAPK, were found to be regulated by AXL in ccRCC through GSEA. Besides, several LncRNA/RBP/AXL mRNA axes were also identified by us to reveal the potential mechanisms of AXL in ccRCC. Subsequent basic researches were required to verify our findings by deep experimental research.

## Supplementary Information

**Additional file 1: Table S1.** Detailed clinicopathological data of these six ccRCC patients.

## Data Availability

The RNA-sequencing data and corresponding clinical information were downloaded from the Cancer Genome Atlas (TCGA) database (https://portal.gdc.cancer.gov/) and the International Cancer Genome Consortium (ICGC) database (https://icgc.org/).

## Abbreviations

ccRCC: Clear cell renal cell carcinoma
OS: Overall survival
TCGA: The Cancer Genome Atlas
ICGC: International Cancer Genome Consortium database
GSEA: Gene set enrichment analysis
TMB: Tumor mutational burden
RCC: Renal cell carcinoma
CML: Chronic myelogenous leukemia
DEGs: Differently expressed genes
FC: Fold change
KEGG: Kyoto Encyclopedia of Genes and Genomes
MSI: Microsatellite instability
NSM: Nonsynonymous mutations
K–M: Kaplan–Meier
DNA methyltransferase 1: DNMT1
DNA methyltransferase 2: DNMT2
DNA methyltransferase 3A: DNMT3A
DNA methyltransferase 3B: DNMT3B
mRCC: Metastatic renal cell carcinoma
CRC: Colorectal cancer
HNSCC: Head and neck squamous cell carcinoma
HCC: Hepatocellular carcinoma
ALDOA: Fructose-bisphosphate aldolase A
TKI: Tyrosine kinase inhibitors
RBP: RNA binding protein
