# Supplementary material for: Prognostic value and immunological role of AXL gene in clear cell renal cell carcinoma associated with identifying LncRNA/RBP/AXL mRNA networks
Source: Cancer Cell Int. 2021 Nov 27;21:625. doi: 10.1186/s12935-021-02322-y (PMC8626946; doi:10.1186/s12935-021-02322-y)
Supplement: Supplementary file 1 — Additional file 1: Table S1. Detailed clinicopathological data of these six ccRCC patients. [file 12935_2021_2322_MOESM1_ESM.docx]

**Table S1**: Detailed clinicopathological data of these six ccRCC patients;

| **ID** | **Gender** | **Age** | **Tumour Size (cm)** | **T** | **N** | **M** | **Stage** | **Grade** |
| --- | --- | --- | --- | --- | --- | --- | --- | --- |
| **ccRCC case1** | Male | 56 | 4.0*4.0*3.0 | T1a | 0 | 0 | I | 1-2 |
| **ccRCC case2** | Male | 42 | 4.5*4.0*3.5 | T1b | 0 | 0 | I | 1-2 |
| **ccRCC case3** | Female | 51 | 8.0*6.0*3.0 | T4 | 1 | 1 | IV | 3-4 |
| **ccRCC case4** | Male | 78 | 6.0*4.5*4.0 | T3b | 0 | 0 | III | 3-4 |
| **ccRCC case5** | Male | 61 | 4.0*4.0*3.5 | T1a | 0 | 0 | I | 1-2 |
| **ccRCC case6** | Female | 65 | 6.0*5.0*5.0 | T1b | 0 | 0 | I | 1 |
